# African Primary Care Research: Writing a research report

**DOI:** 10.4102/phcfm.v6i1.639

**Published:** 2014-06-06

**Authors:** Ian Couper, Bob Mash

**Affiliations:** 1 Department of Family Medicine, University of the Witwatersrand, South Africa; 2 Family Medicine and Primary Care, Stellenbosch University, South Africa

## Abstract

Presenting a research report is an important way of demonstrating one's ability to conduct research and is a requirement of most research-based degrees. Although known by various names across academic institutions, the structure required is mostly very similar, being based on the Introduction, Methods, Results, Discussion format of scientific articles.

This article offers some guidance on the process of writing, aimed at helping readers to start and to continue their writing; and to assist them in presenting a report that is received positively by their readers, including examiners. It also details the typical components of the research report, providing some guidelines for each, as well as the pitfalls to avoid.

This article is part of a series on African Primary Care Research that aims to build capacity for research particularly at a Master's level.

## Abstrait

**Recherche africaine dans le Domaine des Soins primaires: Comment rédiger un rapport de recherche**. La présentation d'un rapport de recherche est une manière importante de montrer sa capacité à faire des recherches et constitue une exigence de la plupart des diplômes basés sur la recherche. Bien qu'elle soit connue sous différentes dénominations dans les institutions académiques, la structure requise est la même, comprenant l'Introduction, les Méthodes, les Résultats, une Discussion des articles scientifiques.

Cet article donne des conseils sur le processus de rédaction pour aider les lecteurs à commencer et à procéder à la rédaction; et aussi les aider à présenter un rapport qui sera reçu positivement par leurs lecteurs, et leurs examinateurs. Il donne aussi en détail les composantes typiques du rapport de recherche, et des directives pour chacune d'entre elles, ainsi que les pièges à éviter.

Cet article fait partie d'une série sur la Recherche africaine dans le Domaine des Soins primaires ayant pour but de développer les capacités de recherche au niveau de la Maitrise.

## Introduction

‘If we knew what it was we were doing, it would not be called research, would it?’, (Albert Einstein).^1^

A research report needs to be written for a Master's degree, or for other degrees with a major research component, and may also be required for funders, research organisations and government or non-government organisations. The purpose of this article is to assist you with producing a research report that is an adequate reflection of the work that you have undertaken. This article is part of a larger series on primary care research and is intended to build research capacity at a Master's level in the African context. More in-depth textbooks are available for those requiring assistance beyond the scope of this article.^2^, ^3^, ^4^

Different academic institutions call these research reports, dissertations, mini-dissertations, assignments, theses or mini-theses. Some universities use the format of a research article as their standard – in this case, students are required to submit their work as an article that is ready to be submitted to a scientific journal, or that may have already been accepted for publication. Whether or not that is the case, the outline is similar and the level of scholarship expected is much the same, although the length of the research article format will be shorter.

In many institutions, ‘research report’ is used for the research component of a Master's programme that is largely course work (such as a MMed degree). However, I have also chosen to use the term research report because of its generic use outside of academia, in order to denote any report that is produced on the basis of completed research. Generally, the format for such reports is fairly standard and, despite some differences, these reports have a common framework, which I will be discussing.

Usually, research output (be it a thesis, dissertation or report) is written using the format: Introduction, Methods, Results, Discussion (IMRD). This is also the structure required by most medical journals for research articles. Commonly, this basic outline is expanded in a research report to include the following sections or chapters (excluding title pages, declaration, contents and acknowledgments):

- Abstract
- Introduction
- Literature review
- Methods
- Results
- Discussion
- Conclusions and Recommendations
- References
- Appendices.

## The process of writing

Undertaking research involves a particular process or set of processes. Often, if you are completing a postgraduate degree (but this may be the case even in many undergraduate degrees), you would have been required to complete a course in research methods in order to enable you to carry out the kinds of research that you were required to conduct. Writing up the research is no less a process, but you may have been taught less about this step.

How, then, do you go about it? The best way is one step at a time! It is easy to say, but harder to do, and many of us have felt daunted by this task. The author, Jane Yolen, reminds us that: ‘[*i*]deas are the cheapest part of writing. They are free. The hard part is what you do with ideas you've gathered’.^5^

Once you have collected and analysed your data and churned out pages of statistics or interview transcripts and themes – what do you do with it? Here is a straightforward way to proceed with writing your report, the key to which is that you do not have to write the report in the IMRD order.

Start first with the methods. In order to do research, a protocol is always required (at least for ethical approval, if not for academic reasons). Thus, you have essentially written the methods section – it is mostly what was in the protocol. You need to remember that you are now describing what you have done, so it is in the past tense and not the future and you need to describe carefully what you actually did, even if you deviated from your protocol, including any obstacles you encountered and how you dealt with them. Then go on to the literature review. Again, you will have done this to some extent in preparing your protocol. Now you can give more space to it. Completing this prepares you well with regard to reviewing and interpreting your results. The introduction could follow as it provides a good lead in and reminds you about the rationale for your research, which is essential for reviewing your results, but many times this is written toward the end. The results are the next logical section, setting out your findings. Move on from this to your discussion, where you reflect on the findings in the light of the literature, your aims and objectives and your understanding of the issues. The conclusions and recommendations round this off. Add the reference list, make a summary (your abstract) and you are done!

Obviously it is a bit more complicated than this, but it is not the mammoth task that many fear. Before starting, it is important to be familiar with the instructions regarding the layout required by your academic institution (or other organisation) that are intended to guide you. Look for a style guide that can give details regarding the technical issues – such as formatting, chapter and/or section layout, headings, line spacing, referencing style – or look at previous examples. Read other research reports – completed reports by Master's students should be available in your institution's library, or an organisation's research reports would be available online or in a central office.

An important principle is to keep focused. It is very tempting to put in lots of extraneous details simply because one can, but sticking to the aim and objectives is crucial. Always ask yourself how any information helps to build the argument (the ‘thesis’) that you are presenting through your report. It is important to know when to stop – when you have written enough, but also when you have read and researched enough and simply need to start writing. The novelist James Rollins said:

‘I think the worst and most insidious procrastination for me is research. I will be looking for some bit of fact or figure to include in the novel, and before I know, I've wasted an entire morning delving into that subject matter without a word written.’^6^

Language is important. You need to be able to communicate your findings clearly to your readers – be they examiners, supervisors, fellow researchers or funders. Again, a style guide is helpful. Simple things, such as writing in full sentences, avoiding jargon, writing out abbreviations in full the first time they are used (in addition to having a glossary), as well as using paragraphs and spacing properly, all make a big difference. Use the spelling and grammar checker provided by Microsoft^®^ Word (set to the language and region you are working in!), but remember that this does not remove the need for careful reading and re-reading of what you have written so as to identify errors. If you are required to produce a report in English and it is not your home language, consider requesting the help of a first-language English speaker to review it, either formally by submitting it to a local language school or independent reviewer and/or editor, or informally by asking a friend or colleague to assist.

## Contents of the report

What, then, should go into each section or chapter of the report? Each component will be discussed briefly, but recognise that the specifics will vary across organisations and institutions.

### Abstract

The abstract is a short summary of the whole report. It should be structured and have clear headings, most commonly using the IMRD format, although often the discussion is replaced with conclusions. It should use different words from the text, but highlight the key issues. It should be less than 500 words long in a research report and half that length in an article format.

### Introduction

The introduction sets out the rationale for the study. This should include an argument for the social value of the study, which has been described in the article on reviewing the literature elsewhere in this series. It should establish the importance and relevance of the study and may also give background information and contextual information about the study. A personal motivation may be included.

### Literature review

The literature review, which is sometimes included in the introduction, particularly when writing in a journal article format, is a whole topic on its own (and a separate article in this series is devoted to conducting a literature review). If it is a stand-alone section, this chapter must also be structured, with an overview of the contents, a description of how the review was conducted, a presentation of the key issues described in the literature and a summary of the major outcomes of the review. Remember that the main purpose of the literature review in this section is to make an argument for the scientific value of the study. This argument should address two main issues: what is already known about the topic and the gaps in that knowledge that your research will be addressing.

You might also include a description of how other researchers have sought to answer the questions you are addressing, thus explaining the methodology you have chosen. In some reports, it might also be necessary to describe the theoretical or conceptual framework for your study. How to do this is described in more detail in the article on reviewing the literature.

Note that direct quotations should be avoided as much as possible and literature should be integrated; a shopping-list style, summarising each article that has been read in a few sentences, is very boring and shows limited engagement with the literature. Also, in most instances it is not necessary to provide detailed statistical results from the literature, except where these are key to understanding your chosen research process.

It is important that literature is not presented uncritically; it needs to be evaluated, with arguments for or against the findings being offered, in relation to the review as a whole.

### Methods

The methods section starts with the aim and objectives (though some institutions expect that to be included in the introduction). Thereafter there must be a step-by-step explanation of the process followed in carrying out the research – including details of the study design, study setting, study population and how people were sampled or selected, data collection tools and processes and data analysis – regardless of the type of research that was undertaken. Note that details of data collection tools (such as survey forms, questionnaires, interview guides or data sheets) should not be included here; they should be referred to and made available in an appendix. Other articles in this series give detailed explanations of the issues to address with regard to different types of study designs and data.

The methods section should include an explanation of the ethical considerations, as is outlined in the first article of this series. In some cases, it may be sufficient to simply state that ethical approval was obtained and to give the reference number and name of the body that approved the research.

When writing this section, you may also comment on any problems encountered in the process of carrying out your research. The limitations of your methods and the implications thereof are usually tackled later, in the discussion section.

### Results

The results section is often the most poorly presented, yet is the core of your report. You need to be selective about how the data is presented – just because you have collected data does not mean it has to be included and much of it can be presented in tables or graphs without needing detailed description. Present the data that explains or relates to your objectives. If you have carried out your research properly, most, if not all, of your data should be relevant, but there is often unnecessary detail. An example is demographics; these are often collected for comparison purposes between groups and do not need to be presented separately as well as in comparisons, or can be summarised in one paragraph or table, rather than presenting each individual characteristic as if they are data with great meaning.

When presenting data in tables or figures, the item should be introduced and referred to in the text before it is inserted. Each table or figure should be numbered sequentially (a separate list of numbers for tables and for figures) and given a title. If necessary, help the reader to understand and make sense of anything in the table or figure that might be confusing or unclear. Resist the temptation to comment, for example, on the significance of your results in the bigger picture; this should be left for the discussion section. However, it is helpful to highlight or synthesise the results into a logical picture for the reader, indicating what the main take-home messages are from the tables or figures.

The most common mistake is misuse of tables and diagrams. Do not repeat everything in the text that is already presented in tables or figures. Likewise, if it can be said clearly in text, no table or figure is needed. If a table or figure is used, then the readers should be left to look at the details for themselves. Note that some institutions require tables and figures to be placed on separate pages, some require them at the end of the results section and some want them interspersed with text.

The presentation of qualitative data is discussed in the article on qualitative data analysis and presentation of results. In essence, the results section will present the themes that have emerged from your qualitative analysis. Your job as the researcher is to describe carefully your interpretation of each theme *in your own words*. Do not leave the quotations to stand alone as an explanation of the theme, although sometimes labelling a theme with the words of a participant can help you to stay close to the original meaning. Each main point should be supported by a single illustrative quotation and you should only use more than one quotation where these show clearly different perspectives or critical nuances in the data. When inserting the quotations it is often helpful to give some indication of the origin of the quotation so that the reader can contextualise the voice and also see that you are using a variety of sources. However, this should not break confidentiality. When there are many themes, they may be presented in a table, with subthemes and quotations linked to these. With complex issues or rich datasets, it is helpful to synthesise the overall picture that has emerged in the form of a conceptual framework or diagram toward the end of the results section.

### Discussion

The discussion section typically has four main focus areas:

- A summary of the key findings that address your aim and objectives.
- A discussion of these key findings in relation to what other researchers have found and to existing practice and policy.
- A discussion of the methodological limitations of your work and how this might influence the interpretation or use of your findings.
- A discussion of your recommendations or the implications of your work for different readership, such as policymakers, future researchers or clinicians.

Researchers often feel a bit helpless at this point, because they have so much data; go back to your objectives and interrogate your data in the light of these. Make sure you are not simply repeating your results in the discussion section. Do not present each of your sets of results systematically in a discussion, with a comment on each. Endeavour rather to synthesise and integrate your findings, interpreting their significance and describing their implications. This is the hardest section, as it requires you to have a good understanding of both your own results and of what is known in the field; and to pull your findings together in order to make sense of them.

The section should include one or more paragraphs on the limitations of your study. Showing you understand the limits of what you have found is important – but be careful not to make sweeping statements; for example, if you say the sample size was too small, explain this statement, because you would have justified your sample size in your methodology. Remember, too, the purpose of different kinds of methodologies – thus, for example, the lack of generalisability of qualitative research is inherent to the method, but you chose the method for other reasons, not for its generalisability.

Recommendations may be part of the discussion or may follow the conclusions as part of that section; either way, the principles are the same. The recommendations must arise from your findings; a common mistake is presenting a bunch of good ideas that you could have listed without even doing the research. Your recommendations should flow directly from your conclusions. Resist the temptation to make a long list – a few well-chosen recommendations are sufficient. And avoid the ‘more research is required’ recommendation, unless you make it very specific.

### Conclusion

‘Conclusions’ consists of exactly what it says – on the basis of your research what do you conclude? What is the bottom line? Conclusions should be related clearly to each of the objectives that you set for the study.

### Reference list

The format of the reference list will depend on the institutional requirements, whether this is a sequential numbering system (such as the Vancouver system), author–date (such as the Harvard system) or another format. The correctness of a reference list is a good indication of how meticulous the researcher is. There are plenty of guides available on the web and your academic institution is likely to have one too. So use one and get it right. Many people use the Microsoft^®^ Word automatic referencing facility for numbered referencing – this is fine, but has drawbacks, such as giving a new number each time a reference is used and not letting you add sections after the reference list. It is much better to use a reference manager such as RefWorks or Endnote (or free software programs, such as Mendeley).

### Appendices

Finally, you will add some appendices. This is where you place your questionnaire or interview guide, if you used such; your ethical clearance certificate; your permission letters to conduct the research; and any extra results that were too detailed to fit into your results section.

## Publication

So now you have completed your research report. There is one final critical step – publication! Your research is not really complete until it has been made available publically through a published journal article. Many would argue that it is an ethical obligation to publish – whether or not you have positive findings.

It is beyond the scope of this article to describe the process of publishing an article, save to give a few brief tips. Firstly, choose your audience and thereby choose your journal. Decide who you are writing for and then tailor your article to the interests of that audience and the requirements of that journal. Secondly, write an abstract first; even if you change your abstract at the end, this forces you to think through and set down what you want to put in your article. Not everything that you covered in your research is relevant. Thirdly, write the article afresh if you are working from a lengthy thesis as you will struggle to shorten it sufficiently. Decide on the focus and write that; if you have carried out good research, it might be worth more than one article. Fourthly, your supervisor is your co-author (unless he or she declines); get feedback from him/her. At the same time, never be afraid to ask another colleague to read a draft article and comment – it is always useful and most people are very willing. Finally, when you submit to a journal, prepare for rejection. Anyone who has published has experienced that (often multiple times). Use it as a way of learning and of improving your article. If and when it is accepted, it is worth celebrating. Disseminate your published article to all those that you know are interested.

If your research report is already written in the format of a journal article, then it may only be necessary to adjust for the specific requirements of the journal to which you will submit it.

Remember, as Greg Daugherty (editor of Money Magazine) said: ‘Rejected pieces aren't failure; unwritten pieces are’.^7^
